# Metabolic Profiling of Fish Meat by GC-MS Analysis, and Correlations with Taste Attributes Obtained Using an Electronic Tongue

**DOI:** 10.3390/metabo9010001

**Published:** 2018-12-21

**Authors:** Ryota Mabuchi, Ayaka Ishimaru, Mao Tanaka, Osamu Kawaguchi, Shota Tanimoto

**Affiliations:** 1 Faculty of Human Culture and Science, Prefectural University of Hiroshima, Hiroshima 734-8558, Japan; xoxo.ayaka.523.xoxo@gmail.com (A.I.); q821004tb@ed.pu-hiroshima.ac.jp (M.T.); 2 Fisheries and Ocean Technologies Center, Hiroshima Prefectural Technology Research Institute, Kure, Hiroshima 737-1207, Japan; o-kawaguchi88559@pref.hiroshima.lg.jp

**Keywords:** GC-MS metabolomics, electronic tongue, taste prediction model, while-fleshed fish, phosphoric acid

## Abstract

To evaluate the taste of ordinary muscle from white-fleshed fish, we used GC-MS metabolomic analysis to characterise the compounds therein, and correlated the obtained data with taste measurements from an electronic tongue. Prediction models using orthogonal partial least squares were produced for different taste attributes, and the primary metabolic components correlated with the taste attributes were identified. Clear differences were observed in the component profiles for different fish species. Using an electronic tongue, differences in tastes were noted among the fish species in terms of sourness, acidic bitterness, umami and saltiness. The obtained correlations allowed the construction of good taste prediction models, especially for sourness, acidic bitterness and saltiness. Compounds such as phosphoric acid, lactic acid and creatinine were found to be highly correlated with some taste attributes. Phosphoric acid in particular showed the highest variable important for prediction (VIP) scores in many of the taste prediction models, and it is therefore a candidate marker to evaluate the tastes of white-fleshed fish.

## 1. Introduction

Fish meat is rich in high-quality protein and lipids. The lipids in fish contain a particularly large quantity of n-3 fatty acids including eicosapentaenoic acid (EPA) and docosahexaenoic acid (DHA), which are thought to have anti-inflammatory and other health benefits [1]. Therefore, fish meat is an important food and its consumption is highly recommended. However, the consumption of fish in Japan is currently falling [2]. One possible reason is the aversion to the flavour of low-quality fish meat. Consequently, correctly evaluating the palatability of fish meat is an important research topic for fisheries and food sciences.

The palatability of a food is commonly perceived with the five senses: gustatory, olfactory, tactile, visual, and auditory. Taste is particularly important among these senses. Therefore, chemical analysis of substances associated with tastes and sensory evaluation are both necessary. To evaluate taste-related substances in fish, many studies measured low-molecular-weight nitrogen compounds: free amino acids, organic bases, guanidino compounds, nucleotides and associated substances, etc. [3,4,5,6]. However, analysing and evaluating the many compounds place high demand on equipment and require appropriate analytical methods, so that the process tends to be very laborious and costly. Meanwhile, metabolic profiling has attracted recent attention as a new technique for evaluating food quality [7,8]. Moreover, correlation analysis using the analytical data as explanatory variables (*x*) and sensory evaluation as response variables (*y*) could specify important components in foods that are perceived as taste by the human sensory system [9]. However, as far as we know, there are no reports of applying this technique to the taste evaluation of fish meat.

The taste of fish meat is very important for assessing its quality such as freshness. Sensory evaluation is generally carried out by human testers. However, in these cases the experimental environments such as panellist selection, individual differences and their fitness must be adequately regulated in order to obtain accurate results. There is also the difficulty in obtaining reproducible results. Research in recent years has led to the development of biosensors that chemically measure the tastes. The electronic tongue as an example taste sensor system can chemically measure tastes in a quantitative manner, based on the electrical potential responses of artificial lipid membranes and taking into account human taste threshold values [10]. Such machine-based evaluation is advantageous compared with the sensory evaluation, which is expensive in terms of training and labour costs of human evaluators. On the other hand, the taste active components of fish meat are mainly water-soluble, low-molecular-weight compounds such as free amino acids, nucleotides, and organic acids. Omission tests on fish and shellfish also clearly indicated that these components are important in constituting the corresponding flavours [3]. Hence, fish meat (and shellfish) extract containing the taste active components could be suitable samples for taste evaluation with an electronic tongue.

Because each taste attribute is given a numerical value by the electronic tongue, these values could be considered as response variables for metabolic profiling. Accordingly, here we report a new technique for evaluating the taste of fish meat, based on metabolic profiling of ordinary muscle from different fish species. The correlation analysis was carried out using orthogonal partial least squares (OPLS), in which comprehensive data for water-soluble, low-molecular-weight compounds measured using GC-MS are used as explanatory variables (*x*), and numerical values for different tastes obtained with the electronic tongue as the response variables (*y*). The relationships between *x* and *y* were statistically analysed by producing prediction models for each taste attribute. Moreover, components that make major contribution to a taste attribute were identified in terms of variable important for projection (VIP) scores for each taste attribute produced by the prediction models. Objective evaluation of taste is a major challenge for the food industry. Because this method makes it possible to describe the taste of fish meat chemically, it is an effective method for objectively evaluating the taste of fish meat.

## 2. Results and Discussion

### 2.1. GC-MS Analysis

**Figure 1:**
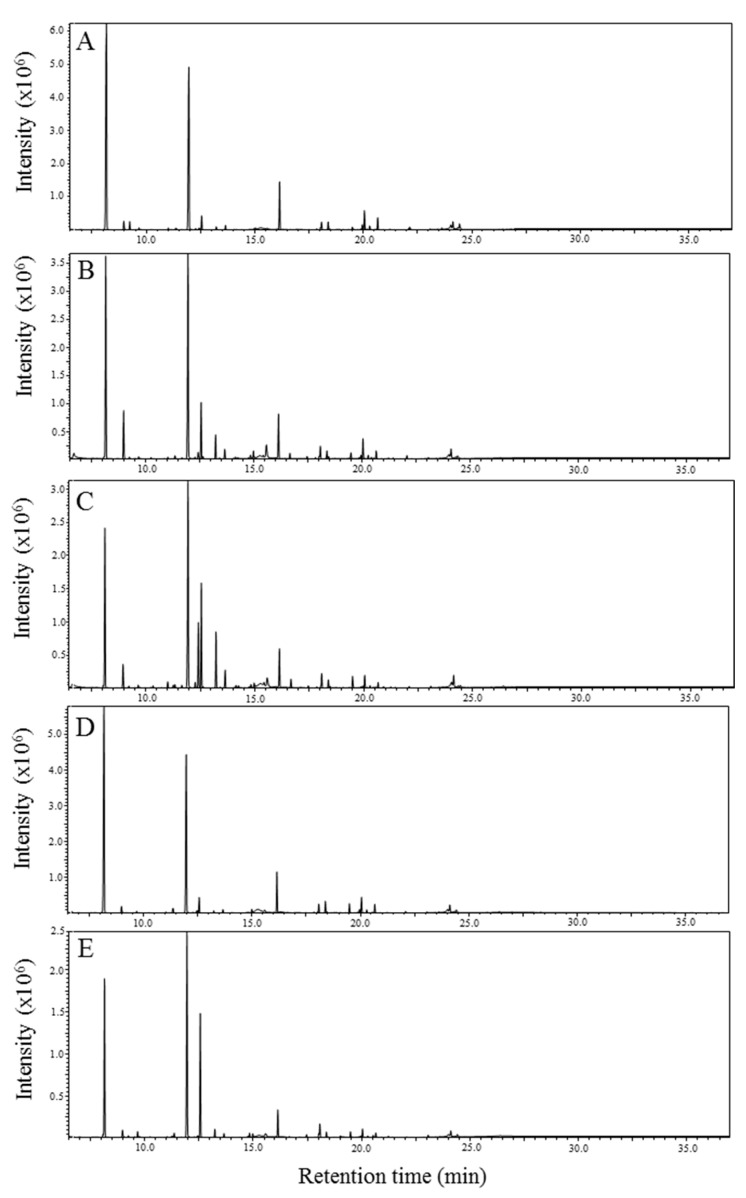
Total ion chromatograms obtained by GC-MS analysis of different fish species. (**A**) *T. modestus*, (**B**) *I. japonicus* (male), (**C**) *I. japonicus* (female), (**D**) *P. major*, and (**E**) *S. marmoratus*.

Typical total ion chromatograms (TIC) obtained by GC-MS analysis of metabolite components extracted from ordinary muscle from each fish species are shown in Figure 1. The shape of the TIC differed slightly among the fish species. The components were detected by comprehensive annotation of the peaks using a commercially available database. The annotated or identified components are presented in Table S1. A total of 136 components were detected. For *Thamnaconus modestus*, *Sebastiscus marmoratus*, *Inimicus japonicus*, and *Pagrus major*, the numbers of detected components were 87, 90, 114, and 99, respectively. These components were classified by their chemical property as follows: 65 sugars including sugar alcohol, sugar acid and amino sugar; 28 amino acids; 18 organic acids; 6 phosphorylated compounds; 4 vitamins and 15 other compounds.

**Figure 2:**
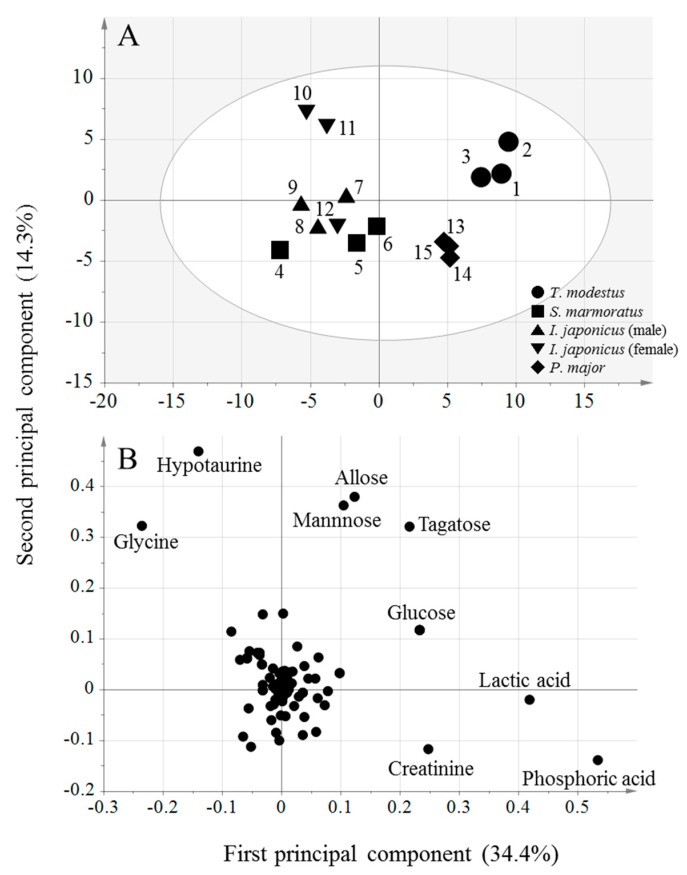
Principal component analysis-X of the component profiles. (**A**) Score plot. Numbers indicated the fish specimens in Table S3. (**B**) Loading plot.

Principal component analysis (PCA-X) was carried out in order to identify differences in the component profiles among the fish species (Figure 2). The first principal component explained 34.4% of the total variance, and the second one explained 14.3%. The cumulative contribution of these two components was 47.7%. The score plots in Figure 2A show that there are differences in the component profiles among fish species. Firstly, in Figure 2A the first principal components for *T. modestus* and *P. major* are positioned in the positive direction, while those for *I. japonicus* and *S. marmoratus* are positioned in the negative direction. This indicates that there are differences in the component profiles between these two pairs of species. In the case of the second principal component, although the differences are not very great, *T. modestus* tends to be positioned in the positive direction, and *P. major* in the negative direction. In the second principal component, part of *I. japonicus* was positioned in the same negative direction as *S. marmoratus*. There were differences between individuals from *I. japonicus*, and in this study no clear difference was found between the species of *I. japonicus* and *S. marmoratus*.

Results for *I. japonicus* and *S. marmoratus* indicated similar component profiles. These two fish species are classified in the same order (Scorpaeniformes), so it is unsurprising that they have comparable component profiles. On the other hand, *P. major* is of order Perciformes and *T. modestus* is of order Tetraodontiformes, and it appears that this considerable phylogenetic difference was reflected in their different component profiles. Therefore, the method here could be an effective technique for discriminant analysis of fish order. While follow-up studies with more fish species are needed in the future, the results above show that it is possible to confirm differences in component profiles among fish species with the experimental conditions in the present study. The loading plot in Figure 2B shows characteristic components in the profiles that reveal differences among the fish species. The figure suggests that hypotaurine and glycine (positioned at the top left of the plot) are characteristic components in *I. japonicus*. Hypotaurine is a precursor in the biosynthesis of taurine [11], while fish and shellfish are rich in taurine. Dark muscles have been reported to include large amounts of taurine, and its content in ordinary muscle also differs depending upon the fish species [12]. However, in this study taurine was not detected experimentally. A possible source of hypotaurine is that cysteine sulfinic acid metabolised from methionine and cysteine can be decarboxylated to hypotaurine by cysteine sulfinic acid decarboxylase (CSD) [11]. The CSD activity varies widely depending on the fish species [13], for example being high in the bluegill and low in the yellowtail. Therefore, the amount of hypotaurine may be an effective marker indicating the different fish species. On the other hand, glycine reacts with arginine in the synthesis of creatine, which is an important energy storage substance in muscles. When adenosine triphosphate (ATP) in muscle is depleted due to strenuous exercise, creatine and ATP are produced from phosphocreatine and ADP to prevent ATP deficiency. Part of the creatine produced by this reaction is converted to creatinine by non-reversible non-enzymatic dehydrogenation. On the loading plot, creatinine, phosphoric acid, and lactic acid are positioned in the lower right, and these components are strongly associated with *P. major*. Moreover, lactic acid is a product of glycolysis and accumulates in the muscle as the result of strenuous exercise. *I. japonicus* is a benthic fish that spends much time hiding in the sea bed and does not normally swim around much, while *P. major* is a more active swimmer in comparison. Since creatinine and lactic acid are components associated with exercise, they are expected to be more associated with *P. major*. On the upper right side of the loading plot, several monosaccharides are shown. These components are strongly associated with *T. modestus*. Characteristic components in fish species obtained by loading plots can suggest associations with their biological differences such as habitats.

### 2.2. Electronic Tongue

In the present study, fish meat was treated with cold water, and the extracted components were submitted to taste sensor analysis. As a result, the present study could easily measure the taste intensities of fish meat. The intensities of each taste obtained by the sensors are shown in Table 1. 

Among the various fish species, taste differences were noted in sourness, acidic bitterness, umami and saltiness. Firstly, there are significant differences in sourness, being stronger in *P. major* and *T. modestus* than in *S. marmoratus* and *I. japonicus* (*p* < 0.05). Acidic bitterness, on the other hand, was significantly weaker in *P. major* and *T. modestus* than in the other two (*p* < 0.05). This result suggests an inverse relationship between these two tastes. The umami was also found to be significantly more intense in *S. marmoratus* and *I. japonicus* (female) compared to *T. modestus* (*p* < 0.05). The saltiness of *S. marmoratus* was significantly lower than the other samples except *I. japonicus* (female) (*p* < 0.05). Umami and saltiness tend to show an inverse relationship. PCA-Y was implemented to visually show the species difference in the taste attributes of fish (Supplementary Figure S1.) PC1 and PC2 could explain 73.0% and 15.3 of the variance, respectively. In the score plot of PCA-Y, the fish species were located at almost the same place as the metabolites from PCA-X. These results suggest a correlation between the metabolites and the taste.

From this, it was presumed that there is a relationship between low-molecular-weight compounds obtained by GC-MS analysis and taste intensities obtained with the taste sensor system.

### 2.3. OPLS Analysis

OPLS was employed in the present study, because it gives models that are easier to interpret and also it is better at screening for marker candidates. The model evaluations obtained by OPLS analysis are shown in Table 2.

*R^2^Y* and *Q^2^Y* values close to 1 mean a better fitting and more accurate model. In general, an *R^2^* value of 0.65 or more and *Q^2^* of 0.5 or more indicate satisfactory ability in quantitative prediction [14,15]. Consequently, in this study models meeting these thresholds were evaluated as statistically significant models. For the tastes of sourness, acidic bitterness, and saltiness, statistically significant models could be produced with all three methods of scaling. For the tastes of irritant, umami, and richness, it was not possible to produce a statistically significant model with pareto-scaling (Par). For bitterness and irritant, a model could only be produced with no-scaling (None). In the None method there is no centring or scaling, while centring and scaling are carried out for unit variance-scaling (UV) and Par, respectively. In UV, each of the variables after centring is divided by its standard deviation, so the result does not reflect quantitative contributions of the variables. On the other hand, with Par, quantitative contributions were considered in the calculations after centring. Targeting only analytical conditions that give statistically significant models (*R^2^Y* ≥ 0.65 and *Q^2^Y* ≥ 0.5), regression equations for the prediction models were produced with the actual measured value *R^2^Y* as vertical axis and the predicted value *Q^2^Y* as abscissa axis (Table 2). When the test of a regression equation for the absence of correlation gives a significance level of 1%, and *n* = 15 as in the present study, a regression equation with Pearson product-moment correlation coefficient (*R^2^*) ≥ 0.64 indicates significant correlation between each taste factor and the metabolites. Therefore, taste prediction models with *R^2^* ≥ 0.64 were considered to be statistically significant. Looking at Table 2, with None it was not possible to find prediction models for any of the tastes. Therefore, it is clearly not a suitable scaling method for producing taste prediction models. On the other hand, prediction models could be produced for some tastes using UV or Par, so centring and scaling with UV or Par should be carried out to produce prediction models. The root mean square errors of estimation (*RMSEE*) and root mean square errors of cross-validation (*RMSE_CV_*) are indices to assess performance of the produced prediction models. Their values in the present study mean the models showed comparatively good precisions (Table 2).

The VIP scores were calculated for each of the *x* variables in the prediction models obtained by OPLS. Many components in each model showed VIP scores ≥ 1.0 (shown in Table S2 as high-VIP components). Sugars show high VIP values in all taste models. Many sugars (for example monosaccharides) are sweet-tasting substances. However, sugar acids like aldonic and uronic acid are sour. In fact, glucuronic acid had a highly positive relationship with sourness (UV). Moreover, acidic bitterness and irritant prediction models show a negative relationship with saccharides. This indicates that differences in saccharide content influence each taste. Therefore, these results suggest that differences in the composition of sugars influence each taste of fish meat.

Analysis with UV successfully detected many compounds with high VIP scores for each of the taste models. On the other hand, analysis with Par found only 1/3 of the compounds detected with UV, but in many cases the VIP values of these compounds were higher than those with UV. With UV, the component ranked first in VIP score was different for each of the tastes. However, with Par, phosphoric acid showed the highest VIP score in all the taste models, and creatinine and lactic acid also showed high VIP scores. The fact that these components were also identified as high-VIP components with UV indicated that they are likely to be effective markers for the taste of fish meat. Phosphoric acid is produced by the breakdown of ATP, which is closely associated with the freshness of fish meat [16]. On the other hand, creatinine and lactic acid are reported to be important in the constitution of the tastes of fish and shellfish in omission tests [3]. For this reason, these components are considered to be candidate markers for evaluating differences in the taste of fish species in this study.

Now, we discuss the high-VIP components for each taste and their association with the tastes. (1) For the taste model of sourness, phosphoric acid and lactic acid, which are acid-tasting compounds, showed positive correlation coefficients. It was therefore suggested that models of sourness were produced by difference in the quantities of acid-tasting substances. (2) In the case of acidic bitterness model, negative correlation coefficients were shown for the acid-tasting compounds (phosphoric acid and lactic acid), creatinine, and many sugars. These observations suggest that acidic and sweet-tasting substances suppress acidic bitterness. (3) In the models of irritant with UV, serine was identified as having the highest VIP score. Although the relationship between serine and irritant is unclear, serine could well constitute a marker for this irritant taste. However, many substances with an astringent taste (e.g., polyvalent metal ions) cannot be analysed by the technique used here. Therefore, specifying components associated with astringency in fish meat requires further investigation. (4) In the models of umami with UV, O-phosphoethanolamine (PEA) showed the highest VIP score. PEA is an important intermediary in phospholipid metabolism, and is widely present in the tissues and bodily fluids of animals. Because it is a non-proteinous free amino acid, it is detected by analysing extractive components of foodstuffs like seafood, and its content varies depending on the foodstuff [17,18]. In fish, the PEA content in the brain of *Perccotts glehni* has been reported to vary with the season [19]. However, there are no reports concerning its relationship with the taste of any food. A positive relationship between PEA and umami in this study suggested that PEA contributes either directly or indirectly to the umami of fish meat. (5) Because saltiness is perceived in the presence of sodium ions, it is difficult to directly evaluate the associated substances by the techniques in this study. However, nicotinamide and other substances that showed a high VIP score in saltiness models with UV might be indirectly associated with saltiness. (6) Aspartic acid, which showed a VIP score of 1.02 in relation to richness, is an amino acid with an umami taste, so it could be associated with richness. The correlation between metabolites and taste attributes was supplemented by a heatmap based on hierarchical clustering analysis (Supplementary Figure S2). The results of the heatmap were similar to those of the OPLS analysis. That is, the metabolites located close to each taste attributes exhibited high VIP values in the taste attribute prediction model (OPLS analysis).

Taste results from the interaction among different chemical substances. Therefore, it can be easily imagined that various components showing high VIP scores for different tastes collectively bring out the flavours peculiar to fish meat. From this reason, the component profile analysis reported here, which comprehensively covers various components with different chemical characteristics, could be an effective new tool for evaluating the taste of fish meat.

## 3. Materials and Methods 

### 3.1. Experimental Samples

Market-size individuals from the species of *T. modestus* (250 ± 28.6 g), *I. japonicus* (male (141 ± 5.9 g) and female (305 ± 103 g)), *S. marmoratus* (541 ± 32.1) and *P. major* (848 ± 114) were used in this study. *T. modestus*, *I. japonicus* and *S. marmoratus* were cultured by feeding with the commercial food Otohime EP (Marubeni Nisshin Feed Co. Ltd., Tokyo, Japan) in the onshore aquaculture cage at the Hiroshima Prefectural Technology Research Institute, Fisheries and Ocean Technologies Center (HiTRI) until use. The *P. major* was cultured by feeding with the commercial food Sakuraou EP (Higashimaru Co. Ltd., Kagoshima, Japan) in the offshore aquaculture tank at HiTRI until use. Three individuals from each species (a total of six for *I. japonicus*) were collected and killed by breaking the bulbar by knife. Immediately after death, each fish was filleted, and the blood-retaining portions were removed as much as possible. The fillets were then stored at −80 °C until analysis. Information about fish species used in this study is summarised in Table S3.

### 3.2. GC-MS Analysis

#### 3.2.1. Pretreatment

The sample preparation was described in a previous publication [20]. In brief, firstly the fillets were freeze-dried and powdered in a mill. Into each 50 mg of powdered sample, mixed solutions of methanol/ultrapure water/chloroform (2.5/1/1 *v*/*v*/*v*, 1 mL) and ribitol (internal reference standard, 0.2 mg/mL, 60 µL) were added. After stirring for 5 min, the mixture was centrifuged (16,000× *g*, 0 °C, 5 min). Ultrapure water (400 µL) was added to 800 µL of the supernatant, followed by stirring for 1 min and then centrifugation (16,000× *g*, 0 °C, 5 min). A 400-µL portion of the supernatant was concentrated for 1 hour using a centrifugal evaporator (CVE-2000, Eyela, Japan), and then freeze-dried overnight. Methoxyamine hydrochloride solubilised with pyridine (20 mg/mL, 50 µL) was added to the freeze-dried sample, and oxime formation was carried out by reacting at 30 °C for 90 min. *N*-methyl-*N*-(trimethylsilyl) trifluoroacetamide with a volume of 100 µL (MSTFA, GL Sciences, Japan) was further added, and trimethylsilylation was carried out by reaction at 37 °C for 30 min. The derivatised samples were submitted to GC-MS analysis.

#### 3.2.2. Analytical Conditions

The GC-MS device was a GCMS-QP2010 Ultra system (Shimadzu, Japan), and the GC column model was Agilent J&W DB-5 (length 30 m, internal diametre 0.25 mm, film thickness 1.00 µm, Agilent Technologies, Santa Clara, CA, USA). The GC oven temperature started at 100 °C, held for 4 min, then increased to 320 °C at the rate of 10 °C/min, and held for 11 min at 320 °C. The injection port temperature was 280 °C. The derivatised sample (1 µL) was injected in split injection mode with a split ratio of 10:1. Helium was employed as the carrier gas, and its linear velocity was kept constant (39.0 cm/s). The purge flow rate was 5 mL/min. Quadrupoles were used for MS mass separation, and electron impact was used for ionisation. The ion source temperature was 200 °C, the interface temperature was 280 °C, and the ionisation voltage was 70 eV. The measurement was carried out in the scan mode in the range of 45–600 *m*/*z*.

#### 3.2.3. Data Processing

The retention time correction of peaks (Retention Index) was carried out based on the retention time of a standard alkane series mixture (C-6 to C-33) using the Automatic Adjustment of Retention Time (AART) function of the Shimadzu GCMSsolution software. The annotation of peaks was performed using commercially available GC/MS Metabolite Component Database Ver.2 (Shimadzu Co. Kyoto, Japan), which contained a mass spectral library. These annotated peaks were detected under the possessing condition of a similarity index of more than 80 and a target ion with confirmation ion ratio of ≥50% in absolute tolerance. Moreover, typically 52 compounds within the annotated peaks were identified by comparison with mass spectra and retention time obtained from standard substance mixture for GC/GC-MS metabolomics (GL Sciences, Tokyo, Japan).

### 3.3. Electronic Tongue

#### 3.3.1. Sample Preparation

Dorsal ordinary muscle (5 g) from fish fillet was homogenised with ACE HOMOGENIZER AM-7 (NIHONSEIKI KAISHA LTD, Tokyo, Japan) at 5000 rpm for 5 min over ice in four fold weight of tap water. After centrifugation (15,000× *g*, 15 min, 4 °C), the supernatant was collected and made up to 70 mL. Of this sample, 35 mL was used for measuring the initial taste and 35 mL for the after taste.

#### 3.3.2. Method of Measurement

The taste was measured by using a TS-5000Z taste sensor system (Insent, Japan) using the method given in an earlier report [21]. Each sample solution was tested using five types of sensors: AAE, CT0, CA0, C00, and AE1. Differences in human perception of taste intensity were estimated based on Weber’s law from the average of three repeated measurements, and the resultant value was taken to be the intensity of each taste attribute. This system detects two types of taste: the initial taste and after taste. In this study, relative potentials obtained from the AAE (umami), CT0 (saltiness), C00 (acidic bitterness), CA0 (sourness) and AE1 (irritant) sensor probes were used to measure selective initial tastes. CPA (change of membrane potential caused by adsorption) values obtained from C00 (bitterness), AE1 (astringency), and AAE (richness) sensor probes were used to measure selective after tastes [21].

#### 3.3.3. Statistical Analysis

The SPSS STATISTICS 24 software (IBM, Armonk, NY, USA) was used for statistical analysis. One-way analysis of variance was used to compare the mean values between fish species for each taste attribute. Attributes that showed significant differences were subsequently tested by Tukey’s multiple comparison test. The significant level was set at 5% (*p* < 0.05).

### 3.4. OPLS Analysis

SIMCA 14 (MKS Instruments, Andover, MA, USA) was used for multivariate analysis. The data sets consisted of: sample name in column 1, fish species in column 2, *y* variables (intensity values for each taste attribute) in column 3, and each corrected peak intensity of the annotated components in columns after the *y* variables. First, PCA-X or Y, as an unsupervised learning analysis without the *y* or *x* variables, was carried out with Par in order to analyse differences in metabolite component or taste attributes profiles of fish species. Next, models for predicting each taste attribute were produced by OPLS analysis using the annotated components as *x* variables, and correlations between each taste attribute (*y* variable) and the metabolic components were analysed. The VIP scores were calculated for the taste attributes for which reasonably precise prediction models were produced, and components with a VIP score ≥ 1 were regarded as metabolite components with a strong relationship with the corresponding taste attributes. Furthermore, OPLS analysis was carried out employing three types of scaling (None, UV, or Par) in order to investigate how the scaling affects the model performance. The heatmap based on hierarchical clustering used MetaboAnalyst 4.0 (http://www.metaboanalyst.ca/) as supplemental OPLS analysis. Using Par scaling, the heatmap was created targeting the top 30 with VIP values obtained with PLS-DA.

## 4. Conclusions

In this study, taste prediction models were constructed based on metabolomic analysis to clarify components important for different tastes in fish meat. Firstly, differences in the component profile from PCA were noted among the four fish species. There were also clear differences between the fish species in sourness, acidic bitterness, umami and saltiness. Moreover, OPLS analysis produced good taste prediction models, especially for sourness, acidic bitterness and saltiness. Various compounds including phosphoric acid, lactic acid and creatinine were specified as important metabolites in the prediction models. Phosphoric acid in particular showed the highest VIP score in many taste prediction models, and therefore it is a candidate marker for evaluating differences in the taste of fish species.

## Figures and Tables

**Table 1:** Intensities of different tastes obtained by electronic tongue for each fish species.

Taste Attributes	Fish Species				
	*Thamnaconus modestus*	*Pagrus major*	*Inimicus japonicus* (Male)	*Inimicus japonicus* (Female)	*Sebastiscus marmoratus*
Sourness	−28.7 ± 1.03 ^a^	−29.5 ± 1.09 ^a^	−34.4 ± 0.92 ^b^	−35.5 ± 1.24 ^b^	−35.3 ± 1.39 ^b^
Acidic bitterness	5.99 ± 0.77 ^a^	5.39 ± 0.70 ^a^	8.61 ± 1.11 ^b^	8.53 ± 0.20 ^b^	8.83 ± 1.13 ^b^
Irritant	−0.76 ± 0.03	−0.83 ± 0.19	−0.11 ± 0.17	−0.30 ± 0.58	−0.70 ± 0.21
Umami	11.0 ± 0.33 ^a^	11.6 ± 0.46 ^ab^	11.8 ± 0.10 ^ab^	12.3 ± 0.28 ^b^	12.4 ± 0.47 ^b^
Saltiness	−17.4 ± 0.48 ^a^	−18.3 ± 0.11 ^a^	−18.1 ± 0.52 ^a^	−18.6 ± 1.66 ^ab^	−20.7 ± 0.42 ^b^
Bitterness	−0.25 ± 0.08	−0.48 ± 0.08	−0.29 ± 0.34	−0.40 ± 0.18	−0.40 ± 0.19
Astringency	−0.27 ± 0.03	−0.27 ± 0.03	−0.25 ± 0.03	−0.25 ± 0.01	−0.28 ± 0.00
Richness	1.13 ± 0.24	1.09 ± 0.25	0.96 ± 0.22	0.93 ± 0.18	0.85 ± 0.24

Values are taste intensity ± S.D. ^a, b^ Mean values within a line with different superscript letters on each fish species differ significantly (*p* < 0.05).

**Table 2:** Evaluation of models obtained by orthogonal partial least squares (OPLS) analysis of each taste attribute. *RMSEE*: root mean square errors of estimation; *RMSE_CV_* : root mean square errors of cross-validation.

Taste	Scaling	A ^a^	N ^b^	*R^2^X*	*R^2^Y*	*Q^2^Y*	*y*	*R^2^*	*RMSEE*	*RMSEcv*
Sourness	UV	1 + 0 + 0	15	0.344	0.907	0.874	0.996x − 0.384	0.91 *	1.02	1.14
	None	1 + 1 + 0	15	0.982	0.989	0.984	0.434x − 18.62	0.27	-	-
	Par	1 + 0 + 0	15	0.587	0.880	0.859	1.000x − 0.034	0.88 *	1.16	1.17
Acidic bitterness	UV	1 + 0 + 0	15	0.343	0.771	0.697	0.983x + 0.246	0.78 *	0.83	0.91
	None	1 + 1 + 0	15	0.982	0.678	0.595	0.729x + 2.095	0.44	-	-
	Par	1 + 0 + 0	15	0.586	0.742	0.698	0.998x + 0.034	0.74 *	0.89	0.89
Irritant	UV	1 + 1 + 0	15	0.471	0.872	0.661	0.972x + 0.006	0.88 *	0.15	0.25
	None	1 + 1 + 0	15	0.981	0.847	0.761	0.882x − 0.062	0.54	-	-
	Par	1 + 0 + 0	15	0.574	0.468	0.362	-	-	-	-
Umami	UV	1 + 1 + 0	15	0.439	0.894	0.663	0.963x + 0.442	0.90 *	0.21	0.41
	None	1 + 1 + 0	15	0.982	0.989	0.985	−0.179x + 13.92	0.08	-	-
	Par	1 + 0 + 0	15	0.582	0.55	0.435	-	-		
Saltiness	UV	1 + 2 + 0	15	0.557	0.963	0.81	0.965x − 0.654	0.96 *	0.29	0.67
	None	1 + 1 + 0	15	0.982	0.988	0.983	−0.152x − 21.42	0.03	-	-
	Par	1 + 1 + 0	15	0.697	0.854	0.599	0.998x + 0.013	0.86 *	0.55	0.83
Bitterness	UV	-	-	-	-	-	-	-	-	-
	None	1 + 1 + 0	15	0.981	0.828	0.745	1.094x + 0.034	0.14	0.19	0.20
	Par	-	-	-	-	-	-	-	-	-
Astringency	UV	1 + 0 + 0	15	0.23	0.537	−0.086	-	-	-	-
	None	1 + 1 + 0	15	0.982	0.991	0.988	0.379x − 0.164	0.12	0.03	0.03
	Par	1 + 0 + 0	15	0.52	0.192	0.0325	-	-	-	-
Richness	UV	1 + 1 + 0	15	0.451	0.864	0.673	0.928x + 0.072	0.87 *	0.09	0.16
	None	1 + 1 + 0	15	0.982	0.979	0.969	1.338x − 0.339	0.55	-	-
	Par	1 + 0 + 0	15	0.576	0.382	0.215				

^a^ A = number of models. ^b^ N = number of samples used in producing models. * Indicates statistically significant differences. UV, unit variance-scaling; Par, pareto-scaling; None, no-scaling.

## Supplementary Materials

The following are available online at http://www.mdpi.com/2218-1989/9/1/1/s1, Table S1: List of metabolites detected on GC-MS analysis, Table S2: Components which show high VIP scores in prediction models, Table S3: Information about the experimental fish samples. Figure S1: Principal component analysis-Y of the taste attribute profiles. Figure S2: Heatmap based on hierarchical clustering analysis visualisation of the differential metabolites and taste attributes in fish species.
