# Supplementary material for: Metabolic Profiling of Fish Meat by GC-MS Analysis, and Correlations with Taste Attributes Obtained Using an Electronic Tongue
Source: Metabolites. 2018 Dec 21;9(1):1. doi: 10.3390/metabo9010001 (PMC6358880; doi:10.3390/metabo9010001)
Supplement: Supplementary file 1 [file metabolites-09-00001-s001.zip › metabolites-402983-suppl/supplementary Figures.pptx]

## Slide 1
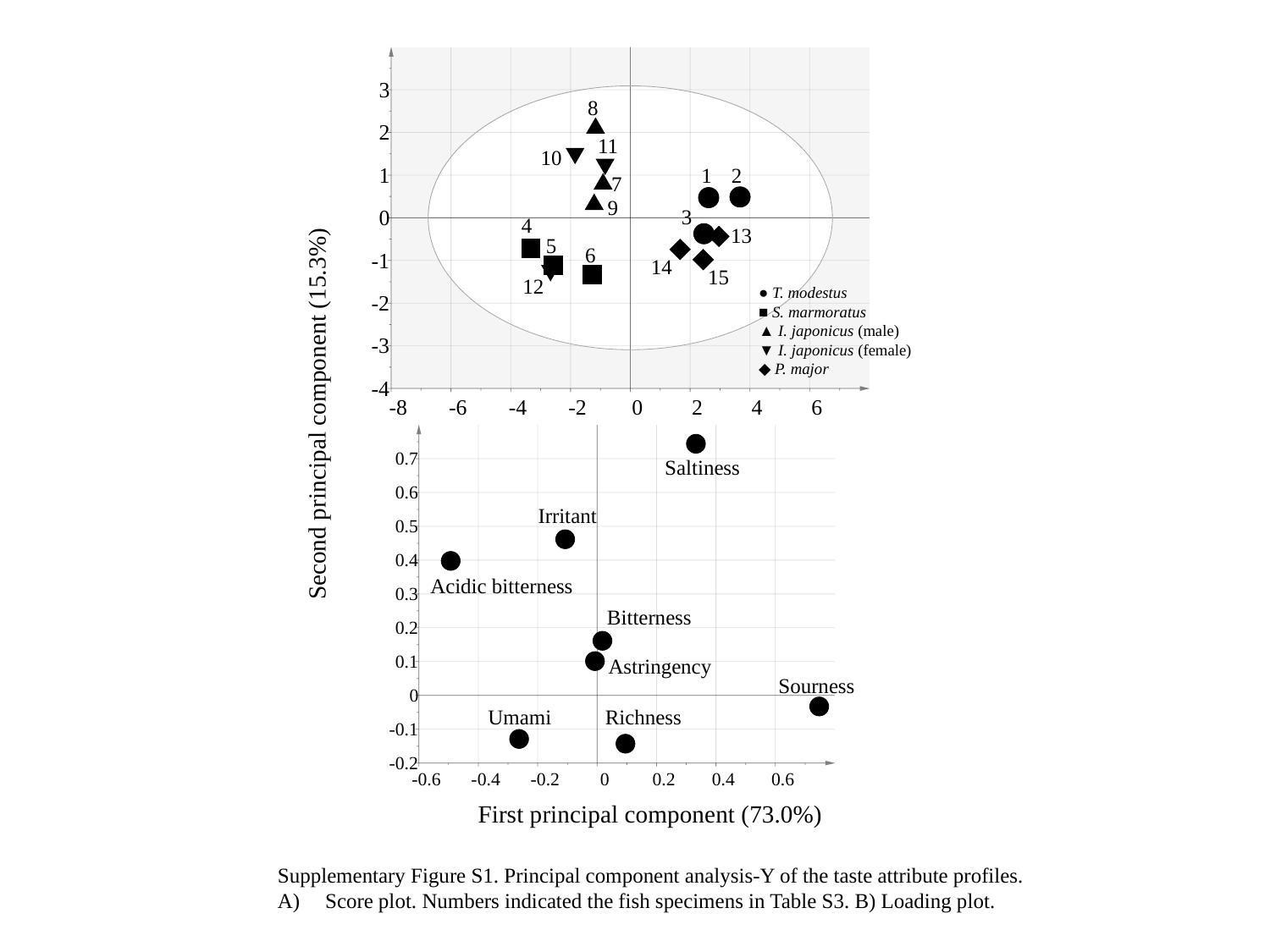

8
11
10
2
1
7
9
3
4
13
5
6
14
15
12
● T. modestus
■ S. marmoratus
▲ I. japonicus (male)
▼ I. japonicus (female)
◆ P. major
Second principal component (15.3%)
Saltiness
Irritant
Acidic bitterness
Bitterness
Astringency
Sourness
Richness
Umami
First principal component (73.0%)
Supplementary Figure S1. Principal component analysis-Y of the taste attribute profiles.
Score plot. Numbers indicated the fish specimens in Table S3. B) Loading plot.

## Slide 2
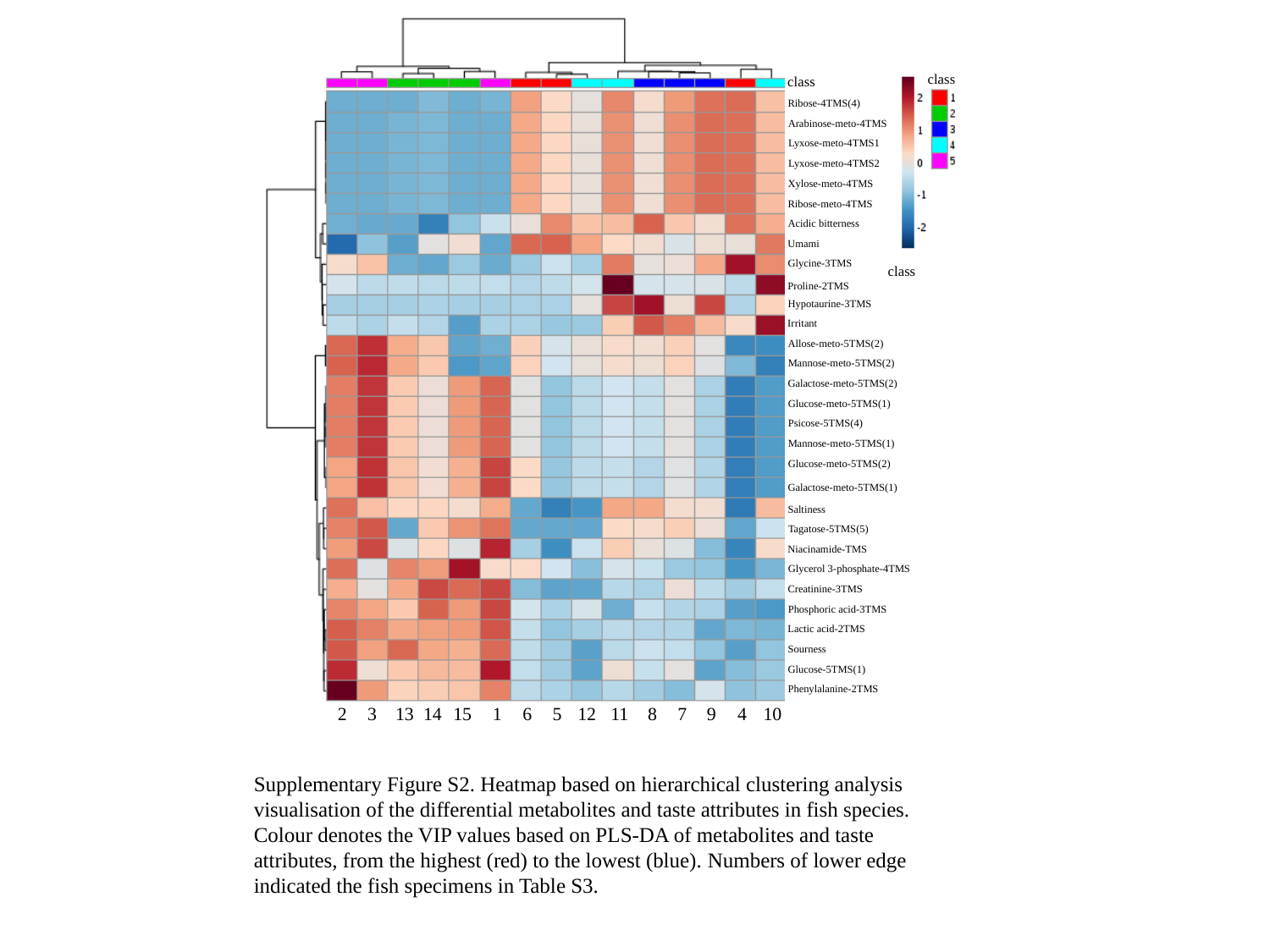

class
class
Ribose-4TMS(4)
Arabinose-meto-4TMS
Lyxose-meto-4TMS1
Lyxose-meto-4TMS2
Xylose-meto-4TMS
Ribose-meto-4TMS
Acidic bitterness
Umami
Glycine-3TMS
class
Proline-2TMS
Hypotaurine-3TMS
Irritant
Allose-meto-5TMS(2)
Mannose-meto-5TMS(2)
Galactose-meto-5TMS(2)
Glucose-meto-5TMS(1)
Psicose-5TMS(4)
Mannose-meto-5TMS(1)
Glucose-meto-5TMS(2)
Galactose-meto-5TMS(1)
Saltiness
Tagatose-5TMS(5)
Niacinamide-TMS
Glycerol 3-phosphate-4TMS
Creatinine-3TMS
Phosphoric acid-3TMS
Lactic acid-2TMS
Sourness
Glucose-5TMS(1)
Phenylalanine-2TMS
2
3
13
14
15
1
6
5
12
11
8
7
9
4
10
Supplementary Figure S2. Heatmap based on hierarchical clustering analysis visualisation of the differential metabolites and taste attributes in fish species. Colour denotes the VIP values based on PLS-DA of metabolites and taste attributes, from the highest (red) to the lowest (blue). Numbers of lower edge indicated the fish specimens in Table S3.
